# Supplementary material for: The interactome of intact mitochondria by cross-linking mass spectrometry provides evidence for coexisting respiratory supercomplexes
Source: Mol Cell Proteomics. 2017 Dec 8;17(2):216–32. doi: 10.1074/mcp.RA117.000470 (PMC5795388; doi:10.1074/mcp.RA117.000470)
Supplement: Supplemental Data [file supp_RA117.000470_133922_0_supp_23978_fzffwf.zip › spectra_annotation/mito_DR_spectra_annotation/300-1-18-1-15-1.pdf]

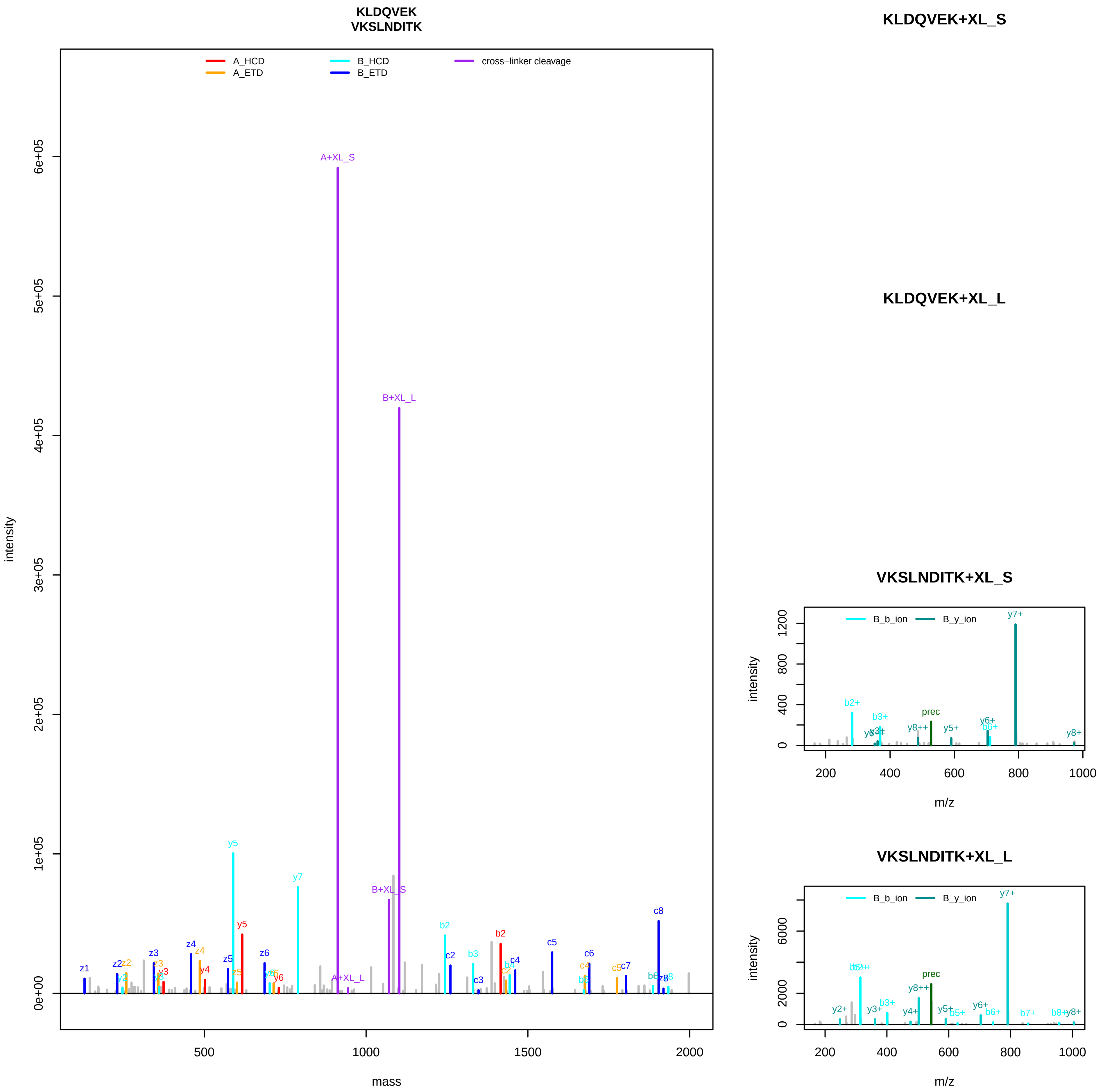

KLDQVEK
VKSLNDITK
A_HCD
A_ETD
B_HCD
B_ETD
cross−linker cleavage
A+XL_S
B+XL_L
B+XL_S
A+XL_L
y5
y7
b2
c8
c5
c6
c7
z1
z2
z3
z4
z5
z6
y3
y4
y6
b3
c2
c3
c4
intensity
mass
0e+00
1e+05
2e+05
3e+05
4e+05
5e+05
6e+05
500
1000
1500
2000
KLDQVEK+XL_S
KLDQVEK+XL_L
VKSLNDITK+XL_S
VKSLNDITK+XL_L
B_b_ion
B_y_ion
prec
y7+
b2+
b3+
y5+
y6+
y8+
y8++
y2+
y3+
y4+
b5+
b6+
b7+
b8+
intensity
m/z
